# Role of Macrophages in Brain Tumor Growth and Progression

**DOI:** 10.3390/ijms19041005

**Published:** 2018-03-27

**Authors:** Elia Guadagno, Ivan Presta, Domenico Maisano, Annalidia Donato, Caterina Krizia Pirrone, Gabriella Cardillo, Simona Domenica Corrado, Chiara Mignogna, Teresa Mancuso, Giuseppe Donato, Marialaura Del Basso De Caro, Natalia Malara

**Affiliations:** 1 Department of Advanced Biomedical Sciences—Pathology Section, University of Naples “Federico II”—via Pansini 5, 80131 Naples, Italy; eliaguadagno84@gmail.com (E.G.); marialaura.delbasso@unina.it (M.D.B.D.C.); 2 Department of Health Sciences, University of Catanzaro “Magna Græcia”—viale Europa, 88100 Catanzaro, Italy; presta@unicz.it (I.P.); maisanodomenico@gmail.com (D.M.); kriziapirrone@gmail.com (C.K.P.); cardillo.gabryella85@gmail.com (G.C.); simona.corrado20@alice.it (S.D.C.); mignogna@unicz.it (C.M.); 3 Department of Medical and Surgical Sciences—University of Catanzaro “Magna Graecia”—viale Europa, 88100 Catanzaro, Italy; annalidia.donato@gmail.com (A.D.); tmancuso@unicz.it (T.M.); 4 Department of Clinical and Experimental Medicine—University of Catanzaro “Magna Graecia”—viale Europa, 88100 Catanzaro, Italy; nataliamalara@unicz.it

**Keywords:** meningioma, macrophage polarization, innate immunity, cancer, glioma, medulloblastoma

## Abstract

The role of macrophages in the growth and the progression of tumors has been extensively studied in recent years. A large body of data demonstrates that macrophage polarization plays an essential role in the growth and progression of brain tumors, such as gliomas, meningiomas, and medulloblastomas. The brain neoplasm cells have the ability to influence the polarization state of the tumor associated macrophages. In turn, innate immunity cells have a decisive role through regulation of the acquired immune response, but also through humoral cross-talking with cancer cells in the tumor microenvironment. Neoangiogenesis, which is an essential element in glial tumor progression, is even regulated by the tumor associated macrophages, whose activity is linked to other factors, such as hypoxia. In addition, macrophages play a decisive role in establishing the entry into the bloodstream of cancer cells. As is well known, the latter phenomenon is also present in brain tumors, even if they only rarely metastasize. Looking ahead in the future, we can imagine that characterizing the relationships between tumor and tumor associated macrophage, as well as the study of circulating tumor cells, could give us useful tools in prognostic evaluation and therapy. More generally, the study of innate immunity in brain tumors can boost the development of new forms of immunotherapy.

## 1. Introduction

The central Nervous System (CNS) was classically considered an “immune-privileged site”, being immunologically inert and separated from the peripheral immune system [1,2].

The Blood-Brain Barrier (BBB) maintains the optimal microenvironment in the CNS and is a major cause of this immune-privilege [3].

Brain endothelial cells lack the expression of leUKocyte adhesion molecules [4]. The lack of these luminal surface molecules prevents the entry of immune cells from the blood into the parenchyma, resulting in a paucity of immune cells in the brain microenvironment [5].

Brain tumors represent a particular case in which BBB is disrupted to different degrees, and therefore acquired and innate immunity may play a role in the development and growth of neoplasia [6]. In cross-talk between tumor cells and the immune system, a very important role is also played by the microglia, the phagocytes traditionally known as resident immune cells in the brain. In the last few years much evidence has been accumulated regarding the relationship between innate immunity and tumors of the central nervous system, mainly in gliomas. Most observations are devoted to study the mechanisms of escape from immunological surveillance in such neoplasms [7,8,9]. Macrophage polarization is currently considered to be a major mechanism of escape from immune control of cancer growth [10,11,12], though the previous paradigm of a strict M1 or M2 state has been largely outmoded [13,14].

For nearly a century, the classification of brain tumors has been based on concepts of histogenesis, based on the idea that tumors can be categorized according to their microscopic similarities with putative cells of origin and their developmental differentiation states. In contrast, the 2016 classification breaks with this old tradition and incorporates well-established molecular parameters into the classification of brain tumors. A grade based on histological features, clinical findings, radiological features, extent of surgical resection, proliferation index values, and genetic alterations is associated with each classified entity, in order to predict the response to therapy and outcome. The genetic profile has to be considered of paramount importance because some genetic changes, such as mutations in the *IDH* gene in diffuse gliomas, have been found to have important prognostic implications [15,16,17] (Table 1).

Despite the large amount of papers concerning the immune response to brain tumors, in the latest WHO classification, the mechanisms and the patterns of immune response are scarcely considered. In this review, we desire to provide an update about the role of macrophages in the growth and progression of brain tumors. Since the data available in the literature are related to the main histotypes, this review will inevitabily focus on gliomas, meningiomas, and medulloblastomas.

## 2. Microglia and Macrophages

In a histological section of normal brain tissue, stained by hematoxylin and eosin, the small dark, elongated nuclei of microglia appear to be ubiquitous. Two types of cells, called microglia, are present in CNS: perivascular microglia and classic resident microglia. Both these components originate from primitive yolk sac macrophages and post-natal hematopoietic progenitors do not significantly contribute to microglia homeostasis in the adult brain, as previously shown in mice [18]. Antibodies directed against scavengers receptors, such as CD68 and CD163, could be used to differentiate microglia and other types of macrophages from non-macrophagic normal and pathological cells of CNS.

The expression of the CD68 receptor, which mediates the recruitment and activation of macrophages, has been widely accepted as a marker for both monocytes and tissue macrophages [19]. It is a member of the lysosomal/endosomal-associated membrane glycoprotein (LAMP) family and localizes to these organelles. Similarly, CD163 is a marker of brain phagocytic cells (microglia), perivascular, and meningeal macrophages, which form flattened, elongated cells, which are adjacent to vessels [20,21,22]. CD163 is a glycoprotein belonging to class B of the scavenger receptor cysteine-rich superfamily, and many authors suggest it as a M2 macrophage-specific marker.

Damage to nervous tissue triggers a macrophage response that serves to clear nonviable debris, creating a particular microenvironment that directs the acquired immune response. All of the macrophage types produce several cytokines like TNF, IL-1, IL-6, IL-8, and IL-12 that influence lymphocyte activation, proliferation, and generation of effector cells. Importantly, macrophages are one of the few cell types that function as antigen-presenting cells. Both pools of autochthonous tissue microglia and the diapedetic monocytes are sources for activated macrophages. Probably the recruitment of blood monocytes plays a predominant role in large lesions, but the supply of indigenous cells is sufficient for little insults [23].

## 3. Gliomas

### 3.1. Tumor Associated Macrophages and “Malignant” Macrophages in Gliomas

Tumor Associated Macrophages (TAMs) derive from circulating macrophages and/or from microglia, but one might ask if a microglioma could be considered to be a real entity in the spectrum of glial tumors. Rare cases of brain neoplasms composed of cells with extremely long, slender and twisted nuclei were described [24]. Recently, new immunohistochemical markers, though not strictly specific, like Ionized calcium-binding adapter molecule 1 (Iba1), have been useful to detect new cases of this putative rare entity [25]. Despite such findings, a neoplasm called “microglioma” is not at present recorded in the WHO classification. 

According to an intriguing hypothesis, “neoplastic macrophages” might be present in glioblastomas (GBMs).

The expression of CD68 antigen has been assessed in glioma tissues around areas where necrosis and foamy cells were absent, since both of the elements could represent a possible cause of inaccurate interpretation. However, astrocytoma cells, according to the same studies, were often CD68 positive [26]. Previous reports [27] stressed the concept that there may be biological properties shared by macrophages and astrocytoma cells, such as phagocytosis and secretion of the same growth and angiogenic factors. These functional similarities may be explained in different ways: (a) by genetic alterations in transformed astrocytes; (b) by fusion of macrophages with astrocytes; or, (c) by a hypothetical mechanism of gene transfer during glioma progression. All of these mechanisms may lead to wrong interpretations while assessing CD68 immunostaining in Malignant gliomas. Many types of human cancers may contain neoplastic cells with mesenchymal/macrophagic properties. Similarly, it has been suggested that neoplastic macrophages/microglia with phagocytic properties, are also present in GBMs, being the most aggressive and invasive cells in such tumors [28]. Cell fusion processes between neoplastic cells and macrophages take place and could be mediated by surface molecules like CD98, previously detected in multinucleated giant cells of GBM [29]. In summary, whereas the existence of a microglial neoplasm has not been ascertained, it is very probable that, in gliomas, macrophages fuse with tumor cells that are generating a set of elements particularly inclined to invading surrounding tissues and enhancing tumor growth.

### 3.2. TAMs and Angiogenesis

The literature data about macrophage infiltration in glial tumors concern mainly neoplasms arising from an astrocytic lineage. The 2016, WHO classification distinguishes gliomas based on the presence or absence of mutations in isocitrate dehydrogenases (*IDH1* and *IDH2*) genes. It has been shown that such mutations may serve as promising prognostic and predictive biomarkers [30,31,32] notwithstanding, till now, that no direct relationship has been established between such genetic alterations and immune response efficacy.

Macrophage deployment is commonly considered to be rare in gliomas unless there has been a treatment for a previously diagnosed neoplasm [33]. Recently, it has been well established that this “histopathological mirage” is a false effect due to an inherent limitation of hematoxylin-eosin analysis; indeed, in gliomas around 30–50% of cells are macrophages or activated microglia and up to 70% of the total cell populations of GBM tissues may be composed of macrophage/microglia [34,35,36].

In radiotherapically treated tumors, macrophages are detectable by immunohistochemical examination, but, areas of residual/recurrent neoplasia contain a smaller number of CD68 positive cells in comParison to other areas [33]. The topographic distribution of TAMs in GBMs, around the perinecrotic areas, into the parenchyma and the perivascular areas has been recently studied by immunohistochemistry. Data showed significative differences in macrophage numbers and a down-regulation of their CD68 receptor expression [37].

It is commonly assumed that hypoxia is one of the major factors acting in the regulation of macrophages phenotype and physiology, in neoplastic context. As the tumor grows, oxygen and nutrient supply, transported via the blood circulation, becomes inadequate. Hypoxic regions can induce angiogenesis by activating hypoxia-inducible factors (HIFs) that upregulate the vascular endothelial growth factor (VEGF). The structure of neoformed vessels in tumors is often abnormal and chronic or cycling intermittent hypoxia is also common in non-necrotic areas [38]. Neoangiogenesis builds gLomeruloid masses of multiple small vessels that represent a classic histological feature in glioblastoma and in other primary CNS neoplasms, such as pilocytic astrocytoma. Another characteristic trait that could be detected is an endothelial multilayered proliferation and thrombosis of small vessels [39].

**Figure 1:**
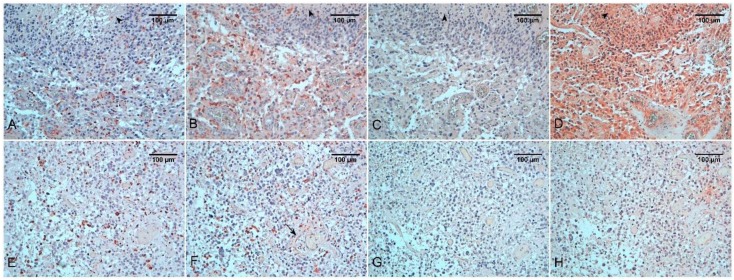
Glioblastoma isocitrate dehydrogenases (IDH)-wild type (**A**–**D**). Immunostaining for CD68 (**A**), CD163 (**B**), inducible nitric oxide synthase (iNOS) (**C**), and hypoxia-inducible factors (HIF-1α) (**D**). Tumor tissue is near a zone of necrosis (arrowhead). Note the strongly positive immunostaining for HIF-1α. Glioblastoma IDH-mutant (**E**–**H**). Immunostaining for CD68 (**E**), CD163 (**F**), iNOS (**G**), and HIF-1α (**H**). In a vessels-rich area macrophages polarization in CD163 positive M2 elements is less evident than in the IDH-wild type case shown above. Elongated, CD163^+^ element, similar to normal perivascular microglia, are present around the vessels (arrow).

**Figure 2:**
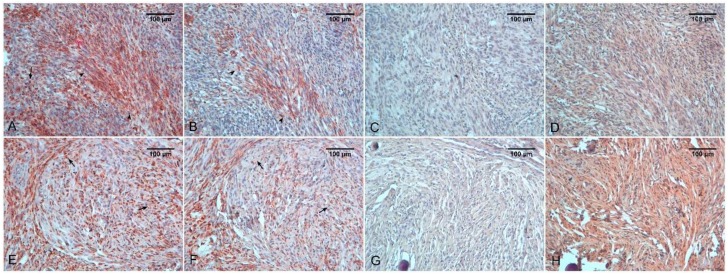
Atypical meningioma (**A**–**D**). Immunostaining for CD68 (**A**), CD163 (**B**), iNOS (**C**), and HIF-1α (**D**). Note a CD68 positive signal in sheet-like growth areas (arrowheads) and in scattered macrophages (arrow). CD163 positivity is lower and immunoreactivity for HIF-1α is very low. Meningothelial meningioma (**E**–**H**). Immunostaining for CD68 (**E**), CD163 (**F**), iNOS (**G**), and HIF-1α (**H**). A more marked positivity for CD163 is evident. Note that CD68 positivity into the whorl is more marked than CD163 staining (Arrows) and strong positivity for HIF-1α.

**Figure 3:**
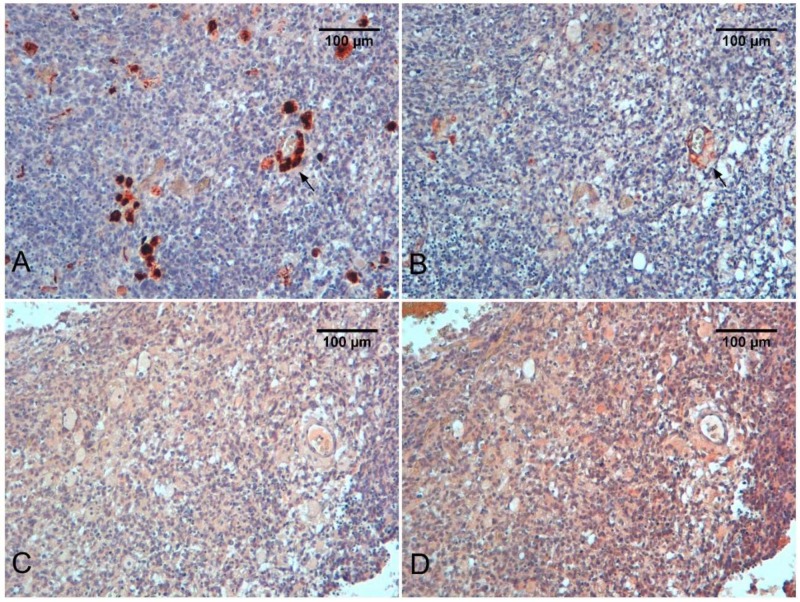
Medulloblastoma. Immunostaining for CD68 (**A**), CD163 (**B**), iNOS (**C**), and HIF-1α (**D**). Note perivascular CD68 positive elements (arrow), probably just recruited from the bloodstream. CD163 is much less expressed in the same elements (arrow).

It is well known that in astrocytic tumors, hypoxia increases dramatically with the tumor grade [40,41,42,43,44,45,46]. It has been demonstrated in mice that HIF-1α availability is important for motility, invasiveness, and homotypic adhesion to endothelial cells of macrophages [36]. It has been highlighted in in vivo experimental models of GBM, that macrophage polarization toward an M2 phenotype (protumoral) and migration into the tumor tissue, is related to the depth of hypoxia. Moreover, it has been shown, by the orthotopic injection of human GBM cells in athymic rats, that when the GBM mass grows, the hypoxia severity increases, and the acquisition of the protumoral phenotype by the macrophagic population could be a consequence [43]. As a rule, in tumors, including brain tumors, hypoxia influences, the macrophage phenotype altering the microenvironment, and finally promoting an alternative cell activation (Figure 1, Figure 2 and Figure 3). Evidence for this mechanism has been obtained both in humans and in other animal models [44,45]. Markers for both alternative and classical macrophage phenotypes are found in TAMs, even if in hypoxic areas there is a more dominant alternative activation [44].

Furthermore, in GBM a complex activation pattern of macrophage/microglia has been suggested by in vitro studies [46]. Moreover, compelling evidence suggests that a real partitioning in M1/M2 categories of macrophages does not exist and it is useless to organize our thinking about microglia [47]. Experimental models have shown that tumor cells can induce HIF-1 in macrophages, upregulating VEGF, and Arginase 1 (*ARG1*) expression via lactate synthesis and generating a molecular signature found in alternatively activated macrophages [14,48]. Recently, it has been shown in gliomas that microglia/macrophages themselves produce VEGF that is important for progression [49,50,51].

Treatment of GBM requires a multidisciplinary approach and includes surgical resection, followed by radiotherapy (RT) and chemotherapic sessions with temozolomide (TMZ). Almost all patients experience tumor progression with almost universal mortality. The median survival from the first diagnosis is less than 15 months, with a two-year survival rate of 26–33% [52,53]. The addition of bevacizumab to standard treatment revealed no increase in overall survival, but improved progression-free survival. Bevacizumab is a monoclonal antibody that binds to the circulating VEGF-A and inhibits its biological activity by preventing the interaction with the VEGF receptor. This causes a reduction in endothelial proliferation and vascular growth within the tumor [54,55].

Interestingly, in turn, VEGF might have a regulatory action on immune system; in the scenario of high grade gliomas it has been shown using the GL261 glioma cell line from C57BL/6 mice. High levels of VEGF induce enlarging tumor volumes and a remodeling of the vascular structures along with a reduced infiltration of microglia/macrophages of around 50%, with a minor accumulation of microglia/macrophages within the perivascular spaces; concomitantly the release of pro-angiogenic factors, such as VEGF declined, suggesting a possible regulatory feedback mechanism [56].

Moreover, in glioblastoma cancer stem cells (GCSCs) and in alternatively polarized TAMs hypoxia stimulates VEGF and stromal cell-derived factor 1 (SDF-1, also known as CXCL12) production [57,58,59]. In mice, it has been demonstrated that SDF-1, acting via CXCR4 receptor, is able to recruit macrophages in the tumor stroma [60]. As a consequence, a therapy targeting SDF-1 could enhance the efficacy of anti-VEGF treatment [61]. Other data showed the capability of GCSCs and TAMs/Tie-2 expressing monocytes to differentiate into endothelial cells in the tumor stroma [62,63,64,65,66,67]. Zhu and collaborators reviewed this theme and perceived that TAMs participate in all stages of angiogenesis, from vasculogenesis and early sprouting to late neovessel stabilization, under the influence of tumor cells [68]. In summary, it is clear that macrophages play a multifactorial role in angiogenetic processes in gliomas, mainly as a source of soluble factors; they represent plastic elements with a multitasking action and so they could be somehow considered as part of the neoplastic population (Figure 1).

### 3.3. TAMs Immunosoppressive Activity

Antigens CD68, CD163, CD200, CD204, F4/80, and IBA-1 are among the most employed markers to identify glioma-associated microglia elements and macrophages (GAMs), while microglia can be distinguished from macrophages through their low expression of CD45 [69,70,71,72]. Glioma-associated microglia and macrophages may establish an immunosuppressive environment for the growing tumor.

Among different brain tumors (astrocytoma, glioblastoma, oligodendroglioma, ependymoma, medulloblastoma, cerebral lymphoma, gangliocytoma, neurocytoma, and germinoma), glioblastoma and anaplastic glioma showed the largest number of mixed cell populations, which consist of macrophages together with branched and ameboid microglia. Ameboid microglia were predominantly found in glial tumors of low malignancy [30].

These microglia elements that adopt an ameboid morphology are thought to reflect the transition to a phagocytic phenotype [69,70]. Since Ml macrophages express high levels of inducible nitric oxide synthase (iNOS; NOS2) to produce nitric oxide (NO) from arginine, this enzyme can be used as a reliable marker for classically polarized macrophages, mainly in confocal microscopy, in double stained CD68^+^/iNOS^+^ cells [31,71,72,73,74,75,76,77,78,79]. Activated microglia cells sometimes express iNOS and so that they may represent a precursor state towards classically activated M1 macrophages or an intermediate polarization condition, as the data obtained in animal models seem to suggest [80,81,82].

Indeed, the mRNA levels of *P2RY12*, a P2 purinergic receptor, and its membrane localization are inversely correlated with increasing malignancy grade of gliomas. In the same way, P2RY12 localization shifts from cytoplasmic in low-grade tumors, to nuclear in high-grade tumors. Cytoplasmic localization of P2RY12 is associated with the expression of typical M1 markers, characteristic of the pro-inflammatory macrophage response. In contrast, the nuclear localization of P2RY12 is predominant in the higher-grade tumors and associated with the expression of M2 marker CD163 [83]. In high grade gliomas, macrophage abundance not only outnumbers that found in low grade entities, but it is probable that the macrophagic population has a different distribution in terms of polarization state. These differences are likely due to the different panel of regulating factors produced by cancer cells. A related phenomenon, at the histopathological level, might be the presence of perivascular lymphocytic infiltrate recordable in diffuse astrocytoma, but not in glioblastoma. So, we need to question ourselves about the role of such residual inflammatory response in growth and progression of astrocytic tumors.

GAMs are not able to induce T-cell proliferation in order to limit tumor growth [84]; on the contrary, macrophages induce a series of changes that cause the production of an immunosuppressive environment, favouring tumor growth. The consequences are: T-cell inhibition, activation, proliferation, and anergy, Regulatory T-cell induction and T-cell inhibition and apoptosis. 

T-cell activation and proliferation in gliomas is inhibited by GAMs in many ways: they can produce immunosuppressive molecules, such as IL-10, TGF-β1 [85,86,87], and IL-6 [88]. IL-10 also down-regulates the HLA-DR (Human Leukocyte Antigen D-Related) expression, a MHC (major histocompatibility complex) class II cell surface receptor, expressed by monocytes. HLA-DR associates with and presents peptidic antigens to the immune system with the purpose of regulating T-(helper)-cell responses. The inhibition of T-cell activation and expansion as well as T cell anergy result also from the absence of co-stimulatory molecules on GAMs, such as CD86, CD80, and CD40. On the other hand, HLA-G, HLA-E and *PTGER2* (Prostaglandin E Receptor 2) expression in GAMs, leads to a reduced activity of NK (Natural Killer lymphocytes cells, Cytotoxic T lymphocytes (CTL), and T-cell proliferation [9].

It is well shown in animal models that a pathway contributing to immunosuppression in GBM involves the cytotoxic T-lymphocyte antigen-4 (CTLA-4), a co-inhibitory receptor, which outcompetes the activity of T-cell costimulatory receptor CD28 for association with CD80 and CD86 [89,90]. Moreover, the inhibitory effects of CTLA-4 act to inhibit T-cell effector function and enhance the inhibitory activity of Tregs in a murine glioma model [91].

The expression of STAT-3 (Signal Transducer and Activator of Transcription 3) could be considered a marker for M2 polarized GAMs and it is activated in GBMs cells as well. The JAK2/STAT-3 pathway regulates many cellular processes in GBM, including cell survival, proliferation, invasion, anti-apoptosis, and immune evasion making STAT-3 a possible target of therapy [92,93].

There are two other important mediators of immune suppression expressed in gliomas. The *FASLG* and *CD274* genes encode for FasL and programmed death-ligand 1 (PDL-1), respectively, two transmembrane proteins interacting with specific receptors and routing signals that inhibit T-cell activation. These interactions are important for immune system regulation and preventing autoimmunity. FasL belongs to the tumor necrosis factor (TNF) family and can be cleaved into a soluble form by metalloproteinases, but also retains the ability to bind FAS receptor and inducing apoptosis [71]. The programmed death-ligand 1 (PDL-1) is a type I transmembrane protein interacting with its receptor inhibits T-cell activation and cytokine production. These genes are expressed both in glioma cells and GAMs, providing an immune escape for tumor cells through cytotoxic T-cell inactivation. [64]. In a mouse model of glioma, both tumor-infiltrating macrophages and microglia were reported to express high levels of PD-L1 and also accounting for 50% of the FasL expressing cells, enough to be considered as a major cause for the induced apoptosis of lymphocytes [94]. Other actors can play opposing roles: data concerning lymphocyte infiltrate and prognosis of gliomas, indicated a beneficial role of immune T cell infiltration inside the tumor in GBM patients, despite multiple tumor immune-escape mechanisms. Indeed, increased CD3+T-cell infiltration is associated with prolonged survival independent of age [95,96].

Instead there is another class of immune cells able to differentiate into immunosuppressive TAMs under the effect of hypoxia and HIF-1α. Such cells are called myeloid derived suppressor cells (MDSC); they express surface markers, such as CD11b and Gr-1, but lack the expression of class II MHC antigens. MDSC are considered to be a mixed population of monocytic and granulocytic cells and they are present in elevated numbers in the bone marrow, blood, and spleen of patients with tumors being associated with poor survival [71].

In conclusion, it is well known that patients with Malignant gliomas suffer from profound local immunosuppression that certainly represents the most important problem to be solved for immunotherapy to be effective. In this context, TAMs play a pivotal role [97]. 

Notwithstanding that such a great mass of data is going to accumulate regarding this theme, the 2016 WHO classification of brain tumors is only based on pathological and molecular characteristics of tumor cells, mainly summarizing previous genetic and pathological research [17,98,99,100,101]. It is probable that, for Malignant gliomas, the formulation of an “immunoscore”, including an immunohistochemical evaluation of GAMs and T-cell infiltrate, will be mandatory in the future and get integrated in the pathological report [102,103].

## 4. Meningiomas

Meningiomas are neoplasms that are arising from meningothelial (arachnoid) cells, usually dura. Even for these neoplasms the latest WHO classification continues to apply criteria solely based on histopathological features and recognizes thirteen histological entities. Although grading and location of the tumor prove to be the most important prognostic factors for meningiomas, an important mass of data have been recently acquired in the molecular and genetic fields. Different histotypes of meningioma have different molecular signatures, and, surprisingly, a similar finding, to a certain extent, is recorded also for CNS location of these neoplasms in which some mutations are more frequent [103,104,105,106,107,108].

CD68^+^ cells are present in meningiomas even if to a lesser extent than in GBM [109], but their potential role in growth and progression of this type of neoplasms has been poorly studied. macrophages are observed in all three grades of malignancy, but these cells are more numerous in the atypical forms [110]. Sometimes infiltration by TAMs may be very pronounced, particularly in cases of meningiomas that carry isolated monosomy 22, where the immune infiltrates also contain greater numbers of cytotoxic T and NK-cells that are associated with an enhanced anti-tumoral immune response. In line with this, the presence of regulatory T cells is usually limited to a small fraction of all meningiomas [111]. Meningiomas with brain invasion show an immune response containing microglial/macrophagic cells at the tumor-brain border that correlates with the disruption of the glial basement membrane and with the malignancy grade of the neoplasm [112].

Most infiltrating immune cells in meningiomas have a phenotype consistent with an HLA-DR^+^ CD14^+^ CD68^+^ CD16^−/+^ CD33^−/+^ monocyte/macrophage lineage origin [113].

Singular tumor subtypes, such as xanthomatous and chordoid meningiomas, may pose particular problems about the role of macrophages.

Sometimes, metaplastic meningiomas may show xanthomatous differentiation consisting of CD68 positive tumor cells; in this subtype, groups of macrophagic elements are present. Similar changes have also been reported in atypical and anaplastic meningiomas. Therefore, it is important to recognize that xanthomatous differentiation can occur in meningiomas, in order to avoid incorrect identification of these cells as macrophages [114]. An intriguing finding in neoplasia is the possible expression of macrophage antigens that may correlate with a more invasive phenotype [115]. CD163 positivity has been found mainly in cells of atypical meningioma [116] (Figure 2A,B,E,F).

Chordoid meningioma (CM) is a rare histotype of meningioma, classified as grade II, which shows a high rate of recurrence following subtotal resection [117,118]. Recently, we have verified the behavior of some innate immunity cells in chordoid meningioma. Our results suggest that macrophages in CMs are mainly in a non-polarized or M2 (alternatively activated macrophages) state and their abundance might be associated with a major potential of relapse; additionally, there is an inverse correlation between the number of mast cells and macrophages [77].

In conclusion, although the literature concerning immune cells in meningiomas is less extensive than that on the same subject concerning gliomas, it can be considered to be highly probable that macrophages also play an important role in the growth and progression of meningothelial neoplasms.

## 5. Medulloblastoma

According to the 2016 WHO classification of brain tumors, medulloblastoma is defined as an embryonal neuroepithelial tumor arising in the cerebellum or dorsal brainstem, presenting mainly in childhood and consisting of densely packed, small round undifferentiated cells with mild to moderate nuclear pleomorphism and high mitotic count [119]. Because of its clinical utility, a molecular classification into four groups has been formulated: group I, Wnt-activated medulloblastoma; group II, SHH (Sonic Hedgehog)-activated medulloblastoma; group 3 and 4 composed of so called non-Wnt/non-SHH tumors [120,121]. The overall prognosis of MB is relatively good when compared to other high-grade tumors, with a five-year overall survival of approximately 70% [122]. The Wnt subgroup shows a favorable prognosis, with only a 10% probability of metastasis via cerebrospinal fluid at diagnosis, and a five-year overall survival of 95–100%. SHH subtype carries a slightly greater risk of metastasis than Wnt, but less than the groups 3 and 4 [123].

Immune cell infiltration in medulloblastoma is known. CD68 positive cells in glioblastoma are significantly higher than those in the general population of all medulloblastomas (Figure 1A,E and Figure 3A). In contrast to the abundant monocytic infiltration, the content of lymphocytes in glioblastomas is surprisingly at a similar level to medulloblastomas [109].

Interestingly, not only do macrophages contribute to the microenvironment of medulloblastoma, but they also show their prevalence in tumors of children in the SHH subgroup. Macrophages express inflammation-related genes, including TAM-related genes as well as CD163 and CSF1R. The increased expression of CD163 and CSF1R suggests that TAMs developed the M2 phenotype, and therefore they are allied with tumor progression [124,125]. Such a datum may suggest possible different therapeutic approaches in different subtypes of medulloblastomas, and also confirm that an evident correlation between subtype prognosis and TAMs infiltration has still to be clarified.

Moreover, immunohistochemical analysis showed that the areas characterized by macrophage infiltration in the SHH tumors corresponded with the areas of increased proliferation; it is further evidenced by positive staining for Ki-67, which is a nuclear marker of cell proliferation, and that is suggestive of a direct paracrine action of TAMs on tumor cells [124].

Medulloblastoma is incurable in one-third of patients despite aggressive standard therapies. The morbidities associated with such treatments candidate medulloblastoma to be addressed with possible immunotherapeutic intervention. For such a neoplasm, the immunological pattern has to be clarified not only in relation to the tumor growth, but also for its possible influence on metastatic seeding trough the cerebrospinal fluid. It was recently demonstrated that a hematogenous route for medulloblastoma leptomeningeal metastases is active, and the C-C Motif Chemokine Ligand 2 (CCL2) production by tumor cells play a pivotal role in such an occurrence. The CCL2/CCR2 axis has been previously implicated in the metastatic process of several adult cancers and it likely plays a role in tumor cells transiting in and out of the blood circulation, through various mechanisms, probably including macrophage recruitment [126,127,128].

## 6. Discussion

The intracranial tumors that we have analyzed in our discussion have differences and similarities regarding their clinical behavior and their biology, also regarding the interrelationship with the immune response, and particularly with the associated macrophagic component. It is well known that gliomas, meningiomas, and medulloblastomas metastasize very rarely in extracranial organs [129,130,131], but medulloblastoma disseminates frequently via the cerebrospinal fluid. TAMs are considered an important factor in the process of metastasization, especially for their ability to induce epithelial-mesenchymal transition in cancer cells of epithelial tumors [132,133,134].

**Figure 4:**
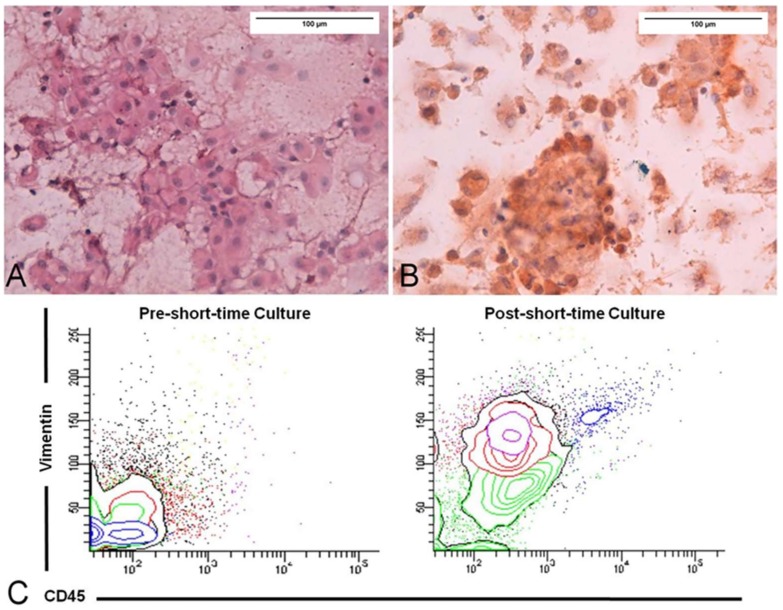
Liquid Biopsy primary short-time culture of a Glioblastoma case: (**A**) Hematossilin/Eosin staining of circulating tumor cells, isolated and cultured in chamber slide for 14 days; (**B**) Immunocytochemical staining using antibody anti-GFAP (Glial Fibrillary Acidic Protein); and, (**C**) Cytometric analysis for vimentinand CD45 antigens expression on circulating cells at time 0 and at day 14 of culture. Scale bar = 100 μm

As shown in a mouse model of HER2^+^ (Erb-B2 Receptor Tyrosine Kinase 2) breast cancer, a permissive effect is also attributed to TAMs for the entry into the bloodstream of circulating tumor cells (CTC) [132]. The presence of CTC in patients with glioblastoma is well documented (Figure 4) and its detection may have a guiding role in diagnosis, prognosis, and therapy [135,136]. Interestingly, CTC from gliomas show features of epithelial-mesenchimal transition [137]. Indeed, in such a specific field, data regarding meningioma and medulloblastoma are less conspicuous. 

Mechanisms by which TAMs facilitate intravasation of tumor cells are mediated via molecular interactions that involve for example a paracrine loop in which epidermal growth factor (EGF), produced by TAMs, increases the invasiveness and migration of cancer cells expressing the EGF receptor (EGFR). Cancer cells, in turn, express CSF1, which acts as a chemoattractant and chemokinetic molecule for CSF1R-expressing TAMs. Equipped with this functional background, tumor cells alternate with macrophages along extra cellular matrix fibers until they reach blood vessels where macrophages facilitate the entry of tumor cells across the vessel wall [138,139]. Moreover, it is known that EGF plays a role in the metastasis of glioblastoma by induction of matrix metalloproteinase-9 in an EGFR-dependent mechanism [140].

An intriguing puzzle appears to be taking shape: we have a heterogeneous group of brain tumors that are not able to produce extracranial metastasis; at least one type of these tumors spreads in the cerebrospinal fluid or releases CTC into the bloodstream. In all of this, TAMs may certainly play a role.

As we said, TAMs inhibit antitumor immunity, removing an obstacle to cancer growth. However, the basic concept underlying the Galon’s immune score [103] may be extended to glioblastoma. Indeed, a significant positive correlation between the increased CD3^+^ and CD8^+^ cellular infiltration into the tumor with improved patient survival has been demonstrated [95]. M2-like TAMs predict poorer prognosis in high-grade glioma and are associated with more aggressive tumors [141]. We consider that a combined analytic approach, which is able to evaluate both lymphocytic and macrophagic components, could help to stratify the patient population more effectively at the prognostic level. Future efforts should focus on establishing more accurate correlations between genetic markers and microenvironmental/functional patterns in order to help therapy setting and formulating a prognostic evaluation.

Regarding the relationship between brain tumor progression and macrophage infiltration, we have shown that macrophages, in higher grade glioma and meningioma, outnumber macrophages that are present in lower grade tumors. 

Moreover, a negative correlation is found between the WHO pathological grades and the expression of M1-type macrophages, whilst, there is a positive correlation between the WHO pathological grades and the expression of M2-type macrophages [142].

Such data are related to a more efficacious recruitment of circulating monocytes and to a specific capacity of alternatively polarize TAMs by higher grade tumors. When macrophages are present into the tumor tissue, they can act as a major factor producing further characteristics typical of higher grade tumors, such as angiogenesis and cell proliferation.

So, one can consider progression as a two step process: cellular progression and progression of the entire tumor tissue. Finally, Innate immunity in brain tumor has to be explored further in order to better understand the mechanisms of progression and growth of these neoplasms and also because immunotherapy may play a larger role in the future therapeutic protocols.

## Figures and Tables

**Table 1:** World Health Organization (WHO) Classification and grades of Main central Nervous System (CNS) tumors.

Tumor Group	Entities	Grade
Diffuse astrocytic and oligodendroglial tumors	Diffuse Astrocytoma, IDH-mutant	II
	Anaplastic astrocytoma, IDH-mutant	III
	Glioblastoma, IDH wildtype	IV
	Glioblastoma, IDH mutant	IV
	Diffuse midline glioma, H3 K27M-mutant	IV
	Oligodendroglioma, IDH-mutant and 1p/19q-codeleted	II
	Anaplastic oligodendroglioma, IDH-mutant and 1p/19q-codeleted	III
Other astrocytic tumors	Pilocytic astrocytoma	I
	Subependymal giant cell astrocytoma	I
	Pleomorphic xanthoastrocytoma	II
	Anaplastic pleomorphic xanthoastrocytoma	III
Ependymal tumors	Subependymoma	I
	Myxopapillary ependymoma	I
	Ependymoma	II
	Ependymoma, RELA fusion-positive	II or III
	Anaplastic ependymoma	III
Other gliomas	Angiocentric glioma	I
	Chordoid glioma of third ventricle	II
Choroid plexus tumors	Chordoid plexux papilloma	I
	Atypical choroid plexus papilloma	II
	Choroid plexus carcinoma	III
Neuronal and mixed neuronal-glial tumors	Dysembryoplastic neuroepithelial tumor	I
	Gangliocytoma	I
	Ganglioglioma	I
	Anaplastic ganglioglioma	III
	Dystplastic gangliocytoma of cerebellum (Lhermitte-Duclos)	I
	Desmoplastic infantile astrocytoma and ganglioglioma	I
	Papillary glioneuronal tumor	I
	Rosette-forming glioneuronal tumor	I
	Central neurocytoma	II
	Extraventricular neurocytoma	II
	Cerebellar liponeurocytoma	II
Tumours of the pineal region	Pineocytoma	I
	Pineal parenghymal tumor of intermediate differentiation	II or III
	Pineoblastoma	IV
	Papillary tumor of the pineal region	II or III
Embryonal tumours	Medulloblastoma (all subtypes)	IV
	Embryonal tumor with multilayered rosettes, C19MC-altered	IV
	Medulloepithelioma	IV
	CNS embryonal tumor, NOS	IV
	Atypcal teratoid/rhabdoid tumor	IV
	CNS embryonal tumor with rhabdoid features	IV
Tumours of the cranial and paraspinal nerves	Schwannoma	I
	Neurofibroma	I
	Perineurioma	I
	Malignant peripheral nerve sheath tumor (MPNST)	II, III or IV
Meningiomas	Meningioma	I
	Atypical meningioma	II
	Anaplastic (Malignant) meningioma	III
Mesenchymal, non-meningothelial tumours	Solitary fibrous tumor/haemangiopericytoma	I, II or III
	Haemangioblastoma	I
Tumors of the sellar region	Craniopharyngioma	I
	Granular cell tumor	I
	Pituicytoma	I
	Spindle cell oncocytoma	I
